# Canine and Human Visual Cortex Intact and Responsive Despite Early Retinal Blindness from *RPE65* Mutation

**DOI:** 10.1371/journal.pmed.0040230

**Published:** 2007-06-26

**Authors:** Geoffrey K Aguirre, András M Komáromy, Artur V Cideciyan, David H Brainard, Tomas S Aleman, Alejandro J Roman, Brian B Avants, James C Gee, Marc Korczykowski, William W Hauswirth, Gregory M Acland, Gustavo D Aguirre, Geoffrey K Aguirre

**Affiliations:** 1 Department of Neurology, School of Medicine, University of Pennsylvania, Philadelphia, Pennsylvania, United States of America; 2 Department of Clinical Studies, School of Veterinary Medicine, University of Pennsylvania, Philadelphia, Pennsylvania, United States of America; 3 Department of Ophthalmology, School of Medicine, University of Pennsylvania, Philadelphia, Pennsylvania, United States of America; 4 Department of Psychology, School of Arts and Sciences, University of Pennsylvania, Philadelphia, Pennsylvania, United States of America; 5 Department of Radiology, School of Medicine, University of Pennsylvania, Philadelphia, Pennsylvania, United States of America; 6 Department of Ophthalmology, University of Florida, Gainesville, Florida; 7 Baker Institute, College of Veterinary Medicine, Cornell University, Ithaca, New York, United States of America; Beth Israel Deaconess Medical Center, United States of America

## Abstract

**Background:**

RPE65 is an essential molecule in the retinoid-visual cycle, and *RPE65* gene mutations cause the congenital human blindness known as Leber congenital amaurosis (LCA). Somatic gene therapy delivered to the retina of blind dogs with an *RPE65* mutation dramatically restores retinal physiology and has sparked international interest in human treatment trials for this incurable disease. An unanswered question is how the visual cortex responds after prolonged sensory deprivation from retinal dysfunction. We therefore studied the cortex of *RPE65*-mutant dogs before and after retinal gene therapy. Then, we inquired whether there is visual pathway integrity and responsivity in adult humans with LCA due to *RPE65* mutations (*RPE65*-LCA).

**Methods and Findings:**

*RPE65*-mutant dogs were studied with fMRI. Prior to therapy, retinal and subcortical responses to light were markedly diminished, and there were minimal cortical responses within the primary visual areas of the lateral gyrus (activation amplitude mean ± standard deviation [SD] = 0.07% ± 0.06% and volume = 1.3 ± 0.6 cm^3^). Following therapy, retinal and subcortical response restoration was accompanied by increased amplitude (0.18% ± 0.06%) and volume (8.2 ± 0.8 cm^3^) of activation within the lateral gyrus (*p* < 0.005 for both). Cortical recovery occurred rapidly (within a month of treatment) and was persistent (as long as 2.5 y after treatment). Recovery was present even when treatment was provided as late as 1–4 y of age. Human *RPE65*-LCA patients (ages 18–23 y) were studied with structural magnetic resonance imaging. Optic nerve diameter (3.2 ± 0.5 mm) was within the normal range (3.2 ± 0.3 mm), and occipital cortical white matter density as judged by voxel-based morphometry was slightly but significantly altered (1.3 SD below control average, *p* = 0.005). Functional magnetic resonance imaging in human *RPE65*-LCA patients revealed cortical responses with a markedly diminished activation volume (8.8 ± 1.2 cm^3^) compared to controls (29.7 ± 8.3 cm^3^, *p* < 0.001) when stimulated with lower intensity light. Unexpectedly, cortical response volume (41.2 ± 11.1 cm^3^) was comparable to normal (48.8 ± 3.1 cm^3^, *p* = 0.2) with higher intensity light stimulation.

**Conclusions:**

Visual cortical responses dramatically improve after retinal gene therapy in the canine model of *RPE65*-LCA. Human *RPE65*-LCA patients have preserved visual pathway anatomy and detectable cortical activation despite limited visual experience. Taken together, the results support the potential for human visual benefit from retinal therapies currently being aimed at restoring vision to the congenitally blind with genetic retinal disease.

## Abstract 2

The study by Samuel Jacobson and colleagues suggests that retinal gene therapy can improve retinal, visual pathway, and visual cortex responses to light stimulation, even after prolonged periods of blindness and in congenitally blind patients.

## Editors' Summary

**Background.:**

The eye captures light but the brain is where vision is experienced. Treatments for childhood blindness at the eye level are ready, but it is unknown whether the brain will be receptive to an improved neural message. Normal vision begins as photoreceptor cells in the retina (the light-sensitive tissue lining the inside of the eye) convert visual images into electrical impulses. These impulses are sent along the optic nerve to the visual cortex, the brain region where they are interpreted. The conversion of light into electrical impulses requires the activation of a molecule called retinal, which is subsequently recycled by retinal pigment epithelium (RPE) cells neighboring the retina. One of the key enzymes of the recycling reactions is encoded by a gene called *RPE65*. Genetic changes (mutations) in *RPE65* cause an inherited form of blindness called Leber congenital amaurosis (LCA). In this disease, retinal is not recycled and as a result, the photoreceptor cells cannot work properly and affected individuals have poor or nonexistent vision from birth. Previous studies in dog and mouse models of the human disease have demonstrated that the introduction of a functional copy of *RPE65* into the RPE cells using a harmless virus (gene therapy) dramatically restores retinal activity. Very recently, a pioneering gene therapy operation took place in London (UK) where surgeons injected a functional copy of *RPE65* into the retina of a man with LCA. Whether this operation results in improved vision is not known at this time.

**Why Was This Study Done?:**

Gene therapy corrects the retinal defects in animal models of LCA but whether the visual pathway from the retina to the visual cortex of the brain can respond normally to the signals sent by the restored retina is not known. Early visual experience is thought to be necessary for the development of a functional visual cortex, so replacing the defective *RPE65* gene might not improve the vision of people with LCA. In this study, the researchers have studied the visual cortex of *RPE65*-deficient dogs before and after gene therapy to see whether the therapy affects the activity of the visual cortex. They have also investigated visual pathway integrity and responsiveness in adults with LCA caused by *RPE65* mutations. If the visual pathway is disrupted in these patients, they reasoned, gene therapy might not restore their vision.

**What Did the Researchers Do and Find?:**

The researchers used a technique called functional magnetic resonance imaging (fMRI) to measure light-induced brain activity in *RPE65*-deficient dogs before and after gene therapy. They also examined the reactions of the dogs' pupils to light (in LCA, the pupils do not contract normally in response to light because there is reduced signal transmission along the visual pathway). Finally, they measured the electrical activity of the dogs' retinas in response to light flashes—the retinas of patients with LCA do not react to light. Gene therapy corrected the defective retinal and visual pathway responses to light in the *RPE65*-deficient dogs and, whereas before treatment there was no response in the visual cortex to light stimulation in these dogs, after treatment, its activity approached that seen in normal dogs. The recovery of cortical responses was permanent and occurred soon after treatment, even in animals that were 4 years old when treated. Next, using structural MRI, the researchers studied human patients with LCA and found that the optic nerve diameter in young adults was within the normal range and that the structure of the visual cortex was very similar to that of normal individuals. Finally, using fMRI, they found that, although the visual cortex of patients with LCA did not respond to dim light, its reaction to bright light was comparable to that of normal individuals.

**What Do These Findings Mean?:**

The findings from the dog study indicate that retinal gene therapy rapidly improves retinal, visual pathway, and visual cortex responses to light stimulation, even in animals that have been blind for years. In other words, in the dog model of LCA at least, all the components of the visual system remain receptive to visual inputs even after long periods of visual deprivation. The findings from the human study also indicate that the visual pathway remains anatomically intact despite years of disuse and that the visual cortex can be activated in patients with LCA even though these people have very limited visual experience. Taken together, these findings suggest that successful gene therapy of the retina might restore some functional vision to people with LCA but proof will have to await the outcomes of several clinical trials ongoing or being planned in Europe and the USA.

**Additional Information.:**

Please access these Web sites via the online version of this summary at http://dx.doi.org/10.1371/journal.pmed.0040230.

- General information on gene therapy is available from the Oak Ridge National Laboratory
- Information is provided by the BBC about gene therapy for Leber congenital amaurosis (includes an audio clip from a doctor about the operation)
- The National Institutes of Health/National Eye Institute (US) provides information about an ongoing gene therapy trial of *RPE65*-Leber congenital amaurosis
- ClinicalTrials.gov gives details on treatment trials for Leber congenital amaurosis
- The Foundation Fighting Blindness has a fact sheet on Leber congenital amaurosis (site includes Microsoft Webspeak links that read some content aloud)
- The Foundation for Retinal Research has a fact sheet on Leber congenital amaurosis
- Find more detailed information on Leber congenital amaurosis and the gene mutations that cause it from GeneReviews
- WonderBaby, information for parents of babies with Leber congenital amaurosis

## Introduction

The childhood-onset incurable human retinal blindness termed Leber congenital amaurosis (LCA) has become a target for in vivo gene transfer because of remarkable success in animal models of several molecular forms [1–5]. The most studied form of LCA is that due to mutations in *RPE65* (*RPE65*-LCA), the critical retinoid (visual) cycle gene that encodes the isomerohydrolase in retinal pigment epithelium (RPE) cells [6,7]. Physiological and biochemical recovery at the level of the retina of RPE65-deficient dogs and mice is dramatic after a single viral-mediated transfer of the *RPE65* gene (for example, [1,2,8,9]). Far less information is available on the details of recovery in postretinal visual pathways [10], and especially cortical visual function [1,11].

Many issues have been addressed during preparation for human ocular gene therapy clinical trials in *RPE65*-LCA patients [12–14], but we remain uncertain about the recovery potential of the visual cortex after prolonged and severe visual deprivation from this congenital retinal defect. The current study addresses this uncertainty with experiments in *RPE65*-mutant dogs and in human *RPE65*-LCA patients. Blood oxygen level–dependent (BOLD) functional magnetic resonance imaging (fMRI) is used to determine if cortical responses to visual stimulation are restored in previously blind *RPE65*-mutant dogs following retinal gene therapy.

The complementary and answerable question prior to human gene therapy in *RPE65*-LCA patients is whether affected individuals have intact visual pathways leading from the defective retina to the visual cortex. Reports of early-onset blind individuals have shown altered visual pathway anatomy [15–17]. To determine the receptivity of cortical substrates for restored retinal input, we evaluated the structure and function of the visual pathways from retina to cortex of young adults with LCA caused by *RPE65* mutations.

## Methods

### Animals and Gene Therapy

A total of eight dogs participated in a total of 12 sessions (Table 1): two normal animals and six *RPE65*-mutant dogs. Animals were examined at a younger (<1 y) or older (1 y or greater) treatment age, crossed with a shorter (1–3 mo) or longer (18–30 mo) duration of treatment prior to MRI scanning. Therapy was delivered by subretinal injection of adeno-associated viral vector carrying the wild-type *RPE65* [1,2]. One of the mutant animals served as a treatment control and received a subtherapeutic dose (viral titer reduced by four orders of magnitude [13]). A total of three mutant animals were studied before and after therapy. All procedures received institutional approval.

**Table 1:**
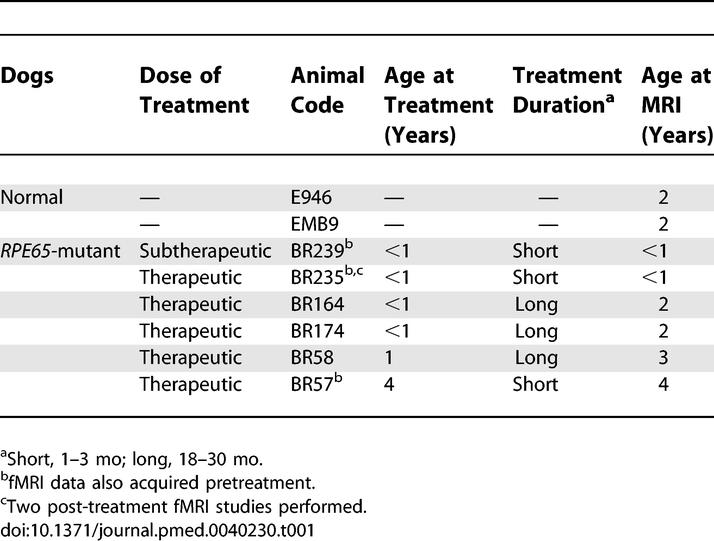
Dogs Studied with fMRI

### Electroretinogram

Dark-adapted dogs, premedicated (acepromazine and atropine) and anesthetized (intermittent IV ketamine), had full-field electroretinograms (ERGs) using published methodology [1,2]. Dark-adapted luminance-response functions were obtained with flash stimuli spanning ~5 log units (−2.5 to +2.2 log scot-cd.s.m^−2^). Threshold and amplitude parameters derived from the ERG were used to compare animals. ERG threshold was defined as the intensity that evoked a criterion (10 μV) b-wave; the amplitude parameter was defined as b-wave amplitude in response to a 2.2 log scot-cd.s.m^−2^ stimulus.

### Transient Pupillary Light Reflex

The direct transient pupillary light reflex (TPLR) was recorded as published [1,10]. TPLR luminance-response functions were elicited with short-duration (0.1 s) stimuli of increasing intensity (green, −6.6 to 2.3 log scot-cd.m^−2^; white, 2.53 log scot-cd.m^−2^); comparisons were made using threshold and amplitude parameters. TPLR threshold was defined as the stimulus intensity that evoked a criterion (0.4 mm) contraction of the pupil diameter (at 0.6 s); TPLR amplitude was defined as the contraction of the pupil diameter (at 0.6 s) elicited by a 0.6 log scot-cd.m^−2^ green stimulus.

### BOLD fMRI Scanning

Prior to scanning, pupils were dilated (topical 1% atropine, 1% tropicamide, and 10% phenylephrine). Anesthesia was with IV ketamine (10 mg/kg) and diazepam (0.28 mg/kg) following SQ atropine (0.05 mg/kg). The anesthetic regimen is comparable to that used for ERG acquisition and preserves optokinetic responses [18]. Neuromuscular blockade (pancuronium 0.1 mg/kg, IV) fixed the eyes in primary gaze; positive pressure ventilation with 100% oxygen was provided. Ventilation and anesthesia were adjusted every 15 min to maintain heart rate and venous blood gas measures in normal range.

Scanning was conducted on a 3.0 Tesla Siemens Trio (http://www.siemens.com) using a standard head coil. Echoplanar images (3 × 3 × 3 mm resolution over 30 slices at TR = 3 s) were obtained during six seven-minute scans, as was a high-resolution (0.4 × 0.4 × 1 mm) MPRAGE anatomical image. Visual stimulation was 21 s of an 18-degree high-contrast reversing (5 Hz) annular checkerboard (0.2–0.6 cycles per degree) with a maximum luminance of 2.8 log cd.m^−2^, alternated with equivalent periods of darkness. The stimulation parameters were guided by a previous canine fMRI study [19].

Data preprocessing and statistical analysis were performed as previously described [20,21]. As the canine hemodynamic response function (HRF) could not be specified a priori, a two-stage analysis was undertaken. First, a Fourier basis set (fundamental frequency and three harmonics) was used to model evoked responses to light stimulation for each animal. The combined explanatory power of these covariates was evaluated with an *F*-test [20].

An average canine HRF (and its first derivative) was then obtained from the evoked response within the lateral gyrus across animals and used in covariate construction for a group analysis. Anatomical registration was accomplished via transformation using SPM2 to a digital canine atlas [22]. No difference in response between the left and reflected right hemisphere was seen at the map-wise level in a group analysis, consistent with binocular successful gene therapy and the visual stimulation protocol we used. Consequently, the data from the two hemispheres were averaged. Group results were displayed upon an inflated cortical atlas, created using the BrainVoyager software (http://www.brainvoyager.com).

Regions of interest (ROIs) within the lateral gyrus, suprasylvian cortex, and lateral geniculate nuclei were defined using the main effect of visual stimulation across all animals. The average amplitude and volume of tissue with a response to visual stimulation (>0.1% change in the canine HRF and first derivative covariates [23]) was obtained for each animal for each ROI. Random-effects comparisons between the groups were conducted with two-sample, one-tailed t-tests.

### Human Participants

*RPE65*-LCA patients (*n* = 6; ages 18–23 y), and control individuals (*n* = 8; ages 20–42 y) participated in the studies. *RPE65* mutations have been previously reported for the six patients [12,24]. All patients were evaluated clinically and with visual and retinal studies using published methods [10,12,25]. An additional 28 control participants (ages 18–23 y) provided whole-brain T1-weighted images for the group analysis of cortical morphology. Informed consent was obtained, and procedures followed institutional guidelines and the Declaration of Helsinki.

### BOLD fMRI Scanning Protocol

A 3.0 Tesla Siemens Trio and an eight-channel head coil were used for scanning. Functional scanning was performed following acquisition of anatomical images (during which the participant remained dark-adapted). A white rectangular screen (subtending 27° × 18°) of uniform luminance and flickering at 5 Hz was presented for 30 s periods, alternated with 30 s periods of darkness. This wide-field unstructured stimulus was chosen to obviate the need for fixation in this population of patients with abnormal eye movements and severe vision loss. Stimulus intensity was varied from low to high over a 7.2 log unit range by sequentially removing neutral density filters from the light path (maximum unattenuated screen luminance, 3.75 log cd.m^−2^). At least two scans were performed at each stimulus intensity.

### Anatomical Image Analysis

Interpial optic nerve diameter was measured on a high-resolution (0.375 × 0.375 × 2.2 mm) T2-weighted anatomical image by one of us (MK), blinded to the assignment of image to population. Measurements from each eye at three locations (1 cm posterior to the globe, anterior to the orbital apex, and at the nerve midpoint) were averaged for each participant. Results for *RPE65*-LCA patients were compared to published norms [26] and controls (*n* = 4).

Voxel-based morphometry [27] was performed upon the T1-weighted MPRAGE images from patients and controls to identify areas of anatomical difference between populations. Preprocessing of anatomical images included automated skull-stripping, nonlinear noise reduction, and tissue segmentation (http://www.fmrib.ox.ac.uk/fsl) [28]. Each brain volume was computationally “warped” to a representative brain, and the log of the determinant of the Jacobian matrix at each point used to index the degree of expansion or contraction [29]. In addition to a whole-brain comparison between the populations, a focused analysis was conducted within anatomically defined ROIs, further constrained by tissue type identified by automated tissue segmentation. Inflated cortical representations were created using commercial software (BrainVoyager).

### Functional Image Processing

Stimulus-induced changes in the BOLD signal were modeled as a “boxcar” covariate, convolved with a population hemodynamic response function [20]. The percentage signal change associated with a level of visual stimulation (derived from the beta value-modeling BOLD signal change relative to the intercept term) was obtained for each voxel for each scan, and the average signal change across population (*RPE65*-LCA patients or control individuals) calculated for each voxel in standard space. As was the case for the canine data, the absence of map-wise differences in hemispheric response allowed us to collapse the data from the two hemispheres to create a single, pseudohemisphere. A second analysis evaluated the degree of functional response observed across a range of stimulus intensities. A region of interest was defined in standard space to include all posterior visual areas (both primary and association cortices). For each level of stimulation the tissue volume that demonstrated a strong response to visual stimulation (>2% signal change) was identified.

## Results

### *RPE65* Gene Therapy Restores Retinal and Subcortical Function to *RPE65*-Mutant Dogs

**Figure 1:**
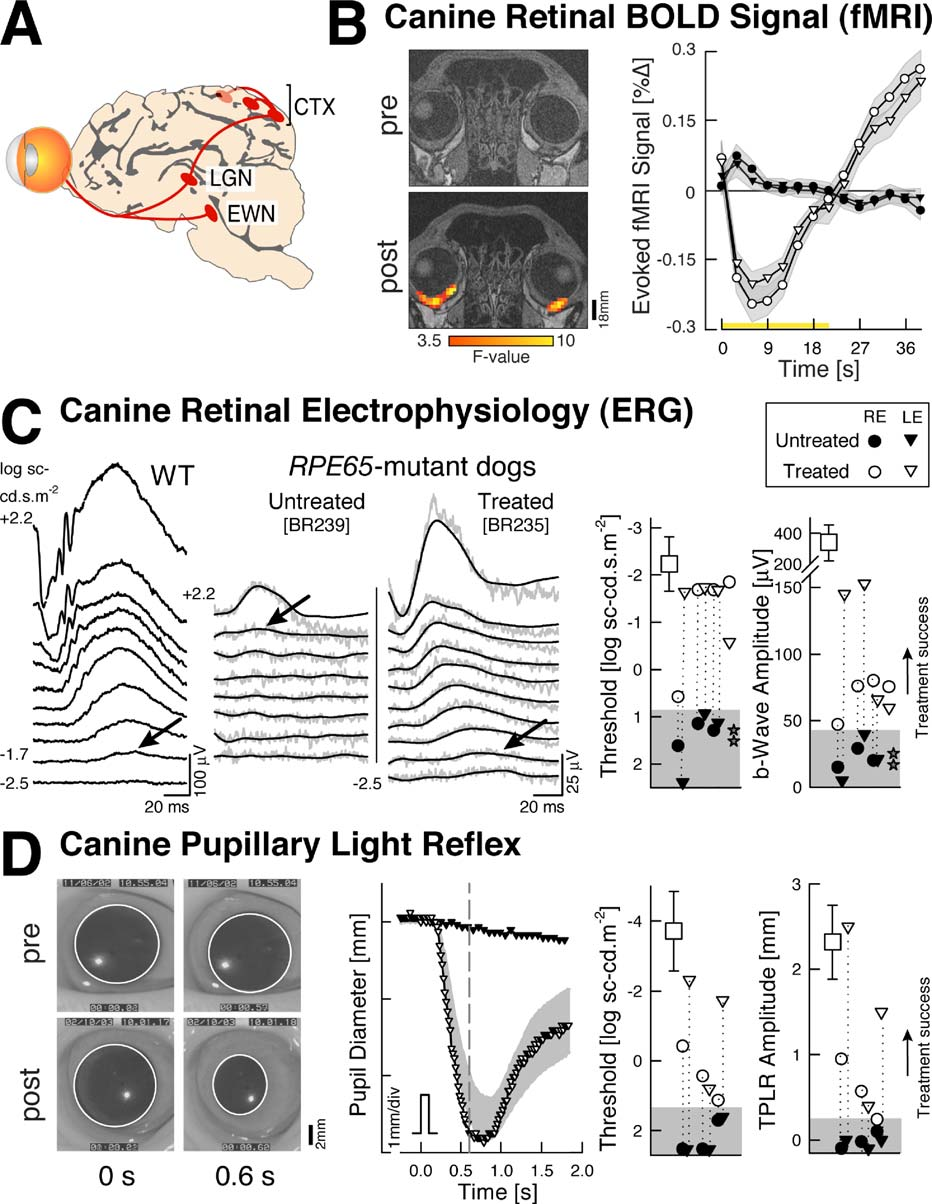
Retinal and Subcortical Responses in *RPE65*-Mutant Dogs Restored with Gene Therapy (A) Visual system structures involved in the measured responses are shown: EWN, Edinger-Westphal nucleus; LGN, lateral geniculate nucleus; CTX, striate and parastriate cortex. (B) Retinal blood flow responses to visual stimulation are presented. Shown is the average BOLD fMRI response (± standard error of mean in gray) to visual stimulation obtained from the eyes of an affected animal (BR235) pre- (filled symbols) and post-treatment (unfilled symbols). After treatment, light stimulation evokes a change in blood flow within the retina, which was absent prior to therapy. The initial negative response of the signal is a consequence of the brief period of stimulation used (21 s) in the face of a very long integration time (>40 s), which has been observed in retinal hemodynamic responses [30]. On the left side are coronal slices through the eye obtained pre- (top) and post-treatment (bottom). Signal responses after treatment (thresholded *F* > 3.5) are seen along the posterior curvature of the globe. Signal loss from susceptibility artifact from frontal sinuses masks any responses from more anterior areas of the eye. (C) Retinal electrophysiology by ERG is shown. Left panels compare waveforms evoked by increasing intensities of light in wild-type (WT) and *RPE65*-mutant dogs (untreated and treated). Raw (gray) and filtered (black) waveforms are displayed for the mutant dogs. Arrows show ERG b-wave thresholds. Right panels show threshold and amplitude parameters in RPE65-mutant dogs 3 mo after treatment (unfilled symbols) compared to wild-type (squares), untreated (filled symbols), and after a subtherapeutic dose (BR239, stars) dogs (vertical dotted lines connect eyes with pre- and post-treatment evaluations; gray region defines mean + 2 SD of the ERG parameter in untreated *RPE65*-mutant eye). (D) Brainstem responses using the TPLR in BR164 pre- and 1 mo post-treatment (video frames show the pupil before and 0.6 s after a 0.6 log scot-cd.m^−2^ stimulus; pupillary margin delineated). Pupillary contraction amplitude and timing in this eye post-treatment (middle panel; unfilled triangles) was within normal limits (gray band). Threshold and amplitude parameters (right panel) show treatment success in *RPE65*-mutant eyes after gene therapy compared to untreated/pretreatment results.

*RPE65*-mutant dogs have severe impairment of retinal and subcortical responses (Figure 1). BOLD fMRI data, acquired through the eye [30] showed no significant response to light stimulation (Figure 1B). ERG response thresholds were elevated by 3–4 log units (Figure 1C), and the normal reflexive contraction of the pupil in response to light was nearly absent (Figure 1D). Retinal gene therapy improved the three measures (Figure 1B–1D). BOLD fMRI signal showed retinal responses to light after treatment. ERG thresholds returned to near normal levels and waveforms increased in amplitude (consistent with a focal area of retina undergoing treatment [2,13]). Pupillary responses to light also recovered following treatment, indicating that brainstem visual pathways can function following retinal gene therapy. To determine whether successful retinal treatment was associated with a recovery of cortical visual responses despite prolonged visual deprivation, we next obtained BOLD fMRI data in wild-type dogs and then *RPE65*-mutant dogs with and without treatment.

### Functional MRI Identifies Striate and Extrastriate Visual Cortex in Normal Canine Brain

**Figure 2:**
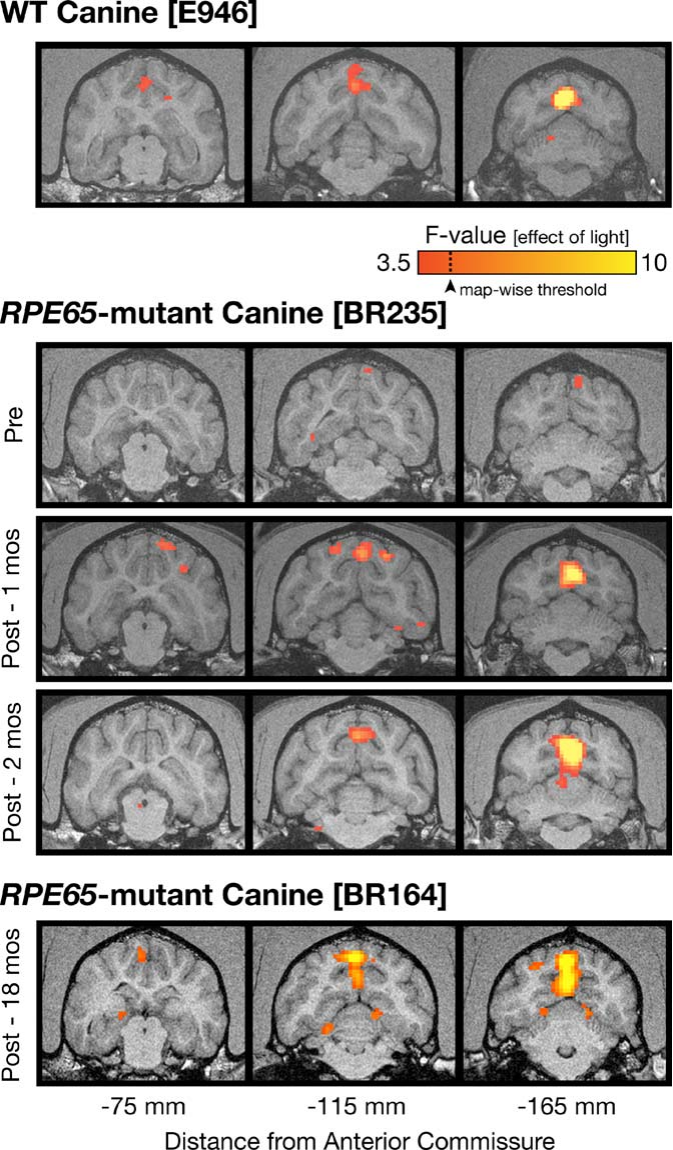
fMRI Responses in *RPE65*-Mutant Dogs before and after Gene Therapy Three coronal slices through the brain are shown, including both the lateral gyrus and extrastriate cortical areas (located within the marginal and ectomarginal sulci). Red and yellow indicate the location of significant responses to light stimulation. Top row: visual responses in a wild-type (WT) dog. Middle three rows: pre- and post-treatment data from an *RPE65*-mutant dog. Post-treatment data were obtained during two separate sessions separated by 1 mo and continue to show WT-like responses in both sessions. Responses within the lateral gyrus pretreatment were seen at a lowered statistical threshold. Bottom row: responses in an animal studied 18 mo after treatment.

**Figure 3:**
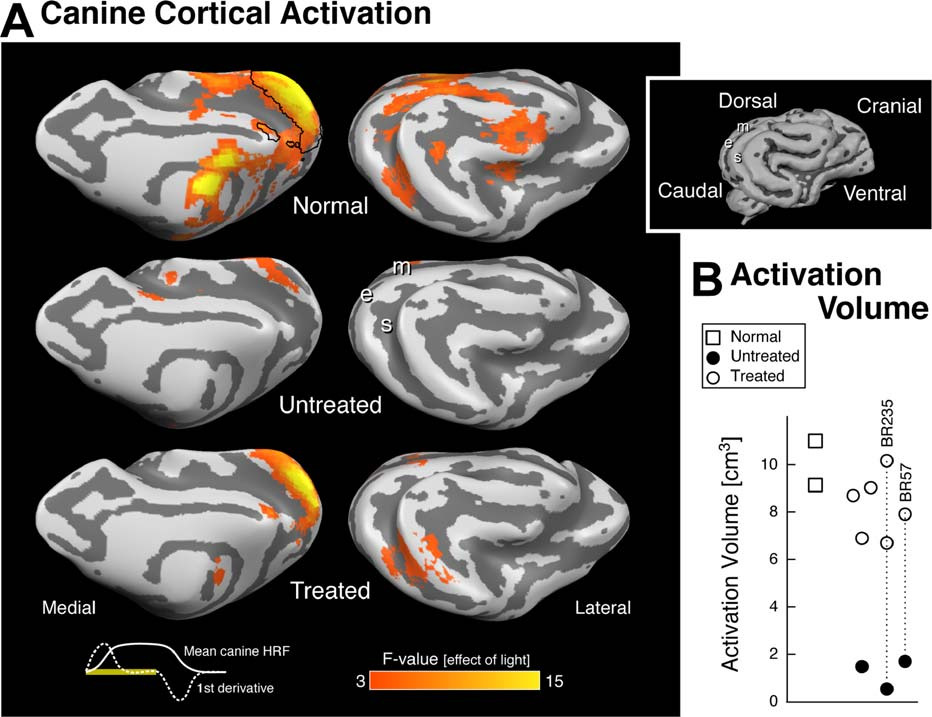
Cortical Responses in *RPE65*-Mutant Dogs (Analyzed as a Group) before and after Treatment Compared to Normal Dogs (A) Areas of cortical activation to visual stimulation are shown in red and yellow on the inflated cortical surface from medial and lateral views (inset shows surface rendering of the initial, folded canine brain). Shades of gray indicate gyral (light) and sulcal (dark) cortex, and the position of three sulci (m, marginal; e, ectomarginal; s, suprasylvian) are marked for reference. In untreated animals (*n* = 3), a small response within the lateral gyrus is present. After treatment (*n* = 5), robust responses in both the lateral gyrus (striate and parastriate cortex) as well as in more laterally located extrastriate areas are seen. The position of the lateral gyrus region of interest examined in (B) is outlined in black on the medial surface of the data from the control animals (*n* = 2). At bottom left the shape of the average canine hemodynamic response (solid white) and its first derivative (dashed white) to 21 s of visual stimulation (yellow bar) are shown. (B) The extent of response within the lateral gyrus region of interest is plotted for treated (unfilled circles), compared to wild-type (squares), and untreated (filled circles) dogs. Vertical dotted lines connect results with pre- and post-treatment evaluations. There is a significant increase in cortical response to light following gene therapy.

Neural responses to light stimulation in wild-type dogs using fMRI under the experimental conditions were first established. FMRI responses to light were observed in normal dogs in posterior cortical areas (Figures 2 and 3). The anatomical site of activity included the lateral gyrus (the posterior, midline structure adjacent to the interhemispheric fissure), as well as a smaller response within the more laterally located ectomarginal and suprasylvian areas. These locations of activity are comparable to the visual areas found in the cat [31,32], with the lateral gyrus containing striate and parastriate cortex (areas 17 and 18), and the distinct laterally located cortical response corresponding to extrastriate visual areas that may be specialized for motion perception [31].

### Retinal Gene Therapy Restores Canine Cortical Visual Responses

*RPE65*-mutant dogs prior to treatment showed no significant cortical or subcortical responses to light stimulation using the conventional map-wise threshold along the visual pathway with fMRI. As the integrated luminance of the fMRI stimulus was within an order of magnitude of the flash ERG stimulus that evoked criterion responses in untreated *RPE65*-mutant dogs (Figure 1C), we asked whether cortical responses were present but too small to detect. Upon lowering statistical thresholds (Figure 2), pretreatment animals showed a weak response eccentrically located within the lateral gyrus; this response was markedly reduced compared to control activation at the same threshold. Group analysis confirmed the presence of minimal but detectable pretreatment responses within the lateral gyrus (Figure 3).

Following successful gene therapy in five animals, significant cortical activation within the lateral gyrus was observed (Figures 2 and 3). In two animals, the extent of the recovered response approached that seen in wild-type controls, despite the limited area of retina that underwent treatment. As the retinal treatment targeted the area centralis [1,2,13], cortical magnification may explain this observation. In two treated animals, significant activity was located within the suprasylvian and ectosylvian cortex, which corresponds to extrastriate cortex in control animals. Subtherapeutic treatment (BR239, Table 1) showed no increase in cortical response (unpublished data)*.*

A group analysis of the five treated animals further illustrates these findings (Figure 3; Table 2). While minimal responses were seen within the lateral gyrus prior to treatment, a marked increase in response was seen following treatment. Within the lateral gyrus (corresponding to striate and parastriate cortex), the treated animals had significantly greater responses to light than were seen in the affected animals prior to treatment (Figure 3B; *t*-test [7 df] = 11.1; *p* < 0.001). The group analysis of treated animals also confirmed increases in the extent of response within the suprasylvian cortex (t[7] = 2.5; *p* = 0.043) and lateral geniculate nucleus (LGN) (t[7] = 3.8; *p* = 0.007). Analyses conducted upon the average amplitude of response within each region, as opposed to spatial extent, yielded similar results (greater response for treated compared to untreated *RPE65* dogs: lateral gyrus t[7] = 5.0, *p* = 0.002; suprasylvian t[7] = 2.3, *p* = 0.056; LGN t[7] = 2.4, *p* = 0.046).

**Table 2:**
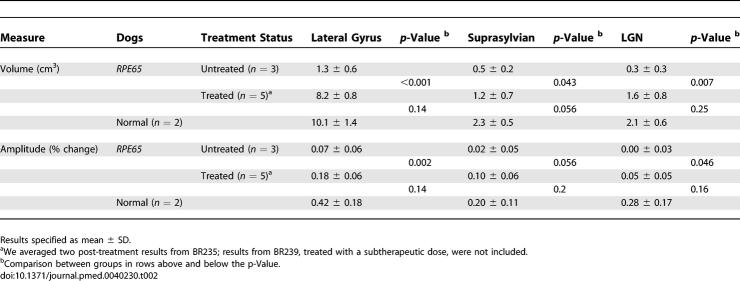
Canine fMRI Cortical Activation

Normal animals showed somewhat greater amplitude and extent of neural responses compared to treated animals (Table 2). The amplitude of response within the lateral gyrus and LGN was significantly greater in control as compared to treated animals in a map-wise fixed effects analysis (unpublished data). There was a trend toward a greater extent of cortical response within the suprasylvian area in the normal animals in the random-effects analysis (Table 2).

Dogs were studied at shorter or longer times following treatment (Table 1). Recovery of cortical responses was observed as soon as 1 mo after therapy, and results were reproducible when studied a second time, 1 mo later (Figure 2). As retinal transgene expression with AAV2 vectors takes approximately two to four weeks following treatment [33], this result indicates that cortical neurons recovered responsiveness quite rapidly following restoration of retinal function. Restored cortical responses were also persistent, in that they were observed in animals treated 18–30 mo earlier (Figure 2). The presence of cortical responses in animals treated at 1 y and 4 y of age begins to answer an important question about effect of age at time of treatment. Further studies in a larger series of animals are warranted.

### Humans with *RPE65*-LCA Show Profound Retinal and Subcortical Dysfunction

**Figure 4:**
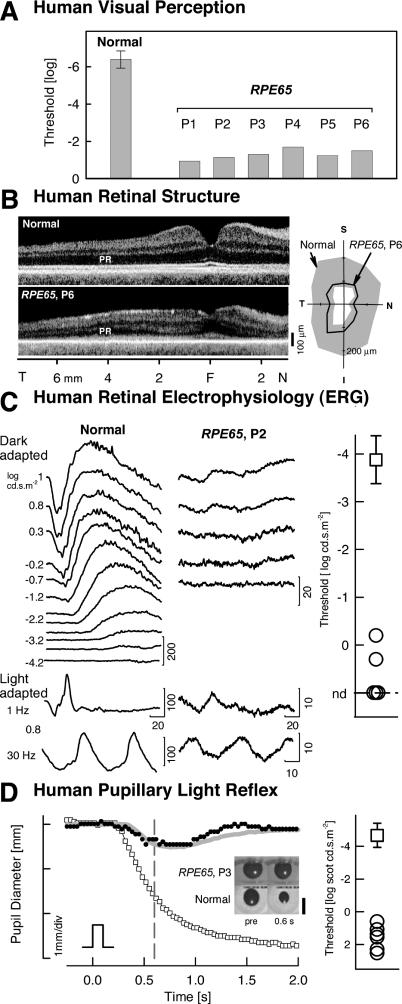
Retinal and Subcortical Dysfunction in Human LCA Due to *RPE65* Mutations (A) Shown are visual thresholds to a full-field stimulus (white, 200 ms) in normal participants (±2 SD) and *RPE65*-LCA patients showing abnormalities of at least 4 log units. (B) Retinal structure by optical coherence tomography is shown. (Left) Cross-sectional retinal images are along the horizontal meridian through the fovea for normal (top) and patient 6 (P6) (bottom). PR, photoreceptor layer; F, fovea; T, temporal; N, nasal. (Right) Polar plot of nerve fiber layer thickness along a circle (diameter 3.4 mm) centered on the optic nerve head is shown. Gray area, normal mean ± 2 SD; S, superior; I, inferior. (C) Retinal electrophysiology by ERG is shown. Left: ERGs to white flashes in the dark- and light-adapted states for patient 2 (P2) and an age-matched normal are shown. Stimulus intensities for dark-adapted responses span 5.2 log units. Patient 2 (P2) shows recordable responses only to the higher stimulus intensities, and amplitudes are severely reduced (note 10-fold change in amplitude scale). Calibration bars are in μV for amplitude and ms for time; stimulus onset is at trace onset. Right: Summary results from patients (circles) show dark-adapted threshold elevations in excess of 3.5 log units from normal (square, mean ± 2 SD). nd, nondetectable. (D) Brainstem responses using TPLR in normal participants and RPE65-LCA patients are shown. Left: Change in horizontal pupil diameter evoked by light stimulus in a normal individual (unfilled squares) compared to patient 3 (P3) (filled symbols) is presented. The smaller and slower pupil response in the patient resembles the normal response to a 5.7 log unit dimmer flash (gray line). Response thresholds were determined at 0.6 s (vertical dashed line). Inset: Images of the pupil before and 0.6 s after a stimulus (2.3 log scot-cd.m^−^2; 100 ms; green). Calibration bar, 6 mm. Right: Summary results from patients (circles) showing threshold elevations in excess of 4 log units from normal (square, mean ± 2 SD).

LCA due to *RPE65* mutations is characterized by marked visual loss from early life. Perceptual thresholds in response to full-field light stimuli are, on average, at least 4 log units elevated (Figure 4A). The retina in *RPE65*-LCA can retain normally organized laminar architecture with a measurable photoreceptor layer into adulthood [12]. Patient 6 (P6), age 23 y, exemplifies the retained photoreceptor layer structure seen in humans with *RPE65* mutations (Figure 4B, left). The retinal output is conducted through axons from retinal ganglion cells, and these axons form the optic nerve. The axon fiber-layer thickness measured in an annular region surrounding the intraocular optic nerve head fell within the normal range in patient 6 (P6) (Figure 4B, right) and in the other patients (unpublished data). Retinal photoreceptor and bipolar cell function, as quantified by the ERG, is severely impaired in animals with RPE65 deficiency [1,2,8], and this was also evident in the retinas of humans with *RPE65*-LCA. Normal human retinas respond to increasing stimulus intensity with increased ERG signal amplitude (Figure 4C). *RPE65*-mutant human retinas (five ERG recordings available) were either nonresponsive to all stimuli (*n* = 3) or only responded at maximal stimulation (*n* = 2). Recordable b-wave (representing bipolar cell activity) amplitudes were, on average, about 3% of normal (8.9 and 13.2 μV for patient 2 [P2] and patient 5 [P5], respectively; normal mean, 440 μV, standard deviation [SD] 92 μV, *n* = 50). ERG thresholds were at least 3.7 log units elevated from normal. Pupillary constriction in response to a short-duration light stimulus quantifies transmission from retina to brainstem nuclei. Pupillometry abnormalities in RPE65-deficient animals have been consistent with the profound retinal defect, showing 4–5 log units of threshold elevation [1,10]. All six patients with *RPE65* mutations had measurable but markedly abnormal pupillary light reflexes (Figure 4D); thresholds ranged from 5.3 to 7.2 log units elevated above mean normal [10].

### *RPE65*–LCA Patients Can Have Near Normal Visual Pathway Anatomy

**Figure 5:**
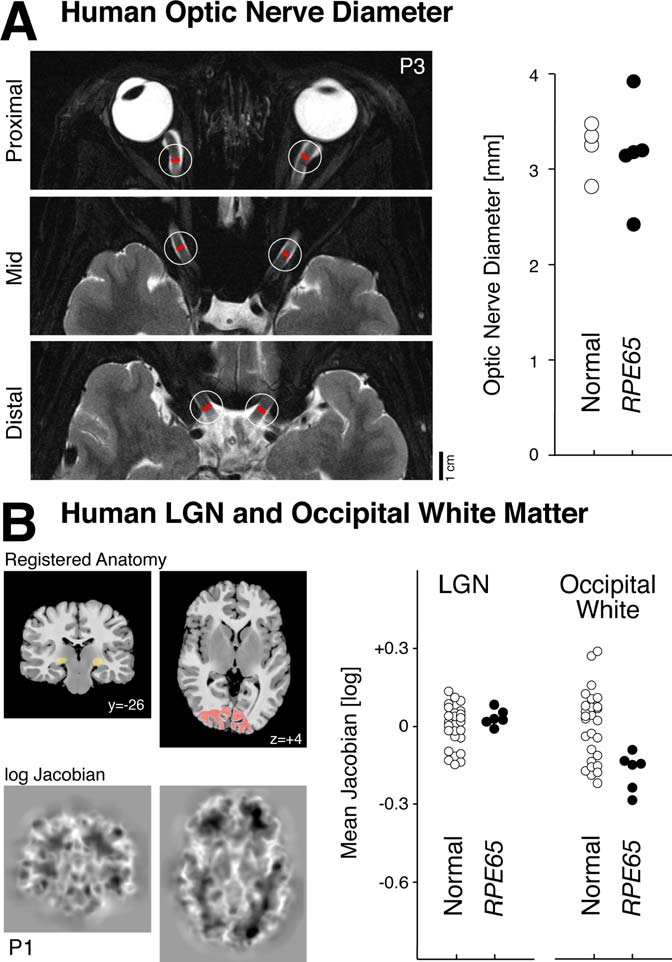
Visual Brain Anatomy in Human LCA from *RPE65* Mutations (A) Interpial optic nerve diameter for patients and controls is shown. Left: Locations of optic nerve measurements were made for patient 3 (P3) upon high-resolution T2-weighted images. The average of six measurements (three from each nerve) were obtained for each participant. Right: Average optic nerve diameters for *RPE65*-LCA patients and controls is shown. No population difference was observed. (B) Whole brain morphometric analysis. Left: The T1-weighted anatomical images from RPE65 patients and controls were warped to a representative template (top row). The (log) determinant of the Jacobian matrix calculated during warping for each participant (bottom row) indexes the degree to which cerebral tissue is smaller or larger than the template image. No differences between patients and controls were present in a whole brain analysis of these measures. A focused analysis was conducted within the LGN and occipital lobe white matter, indicated in yellow and red on the registered anatomy. Also shown is the y- or z-position (mm) of each slice relative to the anterior commissure. Right: The average (log) Jacobian measure within the regions of interest for *RPE65*-LCA patients and controls is shown. Measures were slightly, but significantly, smaller for patients within occipital white matter, indicating relative atrophy.

Does severe early visual deficit in humans with *RPE65* mutations lead to atrophy of the orbital optic nerves and thinning of occipital lobe gray and white matter as reported in forms of early blindness [16,17]? High-resolution (375 μm) images were obtained through the intraorbital optic nerves, and the average interpial diameter was measured (Figure 5A). *RPE65*-LCA patients and age-matched control individuals both had an average optic nerve diameter of 3.2 mm, in agreement with published normal data using high-resolution MRI and histological examination [26].

We next examined if alterations in cerebral anatomy are present in humans with *RPE65*-LCA. The 1-mm resolution whole-brain anatomical images obtained from patients with *RPE65*-LCA were compared to a population of age-matched normal individuals (*n* = 28). The analysis included both cortical gray and white matter as well as subcortical structures (such as the LGN) (Figure 5B). No significant differences between the two populations were found at the map-wise level (*p* > 0.4).

It is possible that focal anatomical differences between the two populations exist, but that our test was insufficiently powered to identify this difference while controlling the false positive rate across the entire brain volume. Given previous reports of differences in early visual areas between early blind patients and controls [16,17] and alterations of the structure of the LGN [17,34], we conducted a more focal test. The LGN was defined in the registered space, and the mean Jacobian measure for this volume of interest was obtained for the controls and patients. No difference in anatomical structure was observed (Figure 5B). Next, the white matter within the occipital lobe underlying early visual areas (i.e., adjacent to the collateral sulcus) was identified. The mean Jacobian measure from this area for two of the *RPE65*-LCA patients fell outside the range of measurements from normal controls (Figure 5B, right panel), and the population mean was slightly more negative (t[31] = 3.0; *p* = 0.005), indicating a small degree of occipital white matter atrophy in the patients as compared to control. While present, this subtle change stands in contrast to the marked reduction in white matter volume seen in patients with early blindness from other causes [17].

### *RPE65* Human Cortex Fully Activates to Suprathreshold Visual Stimulation

Does a profoundly insensitive retina from early life in humans with *RPE65* mutations lead to reduced extent of cortex devoted to visual processing? This would be the prediction from the literature on effects of visual deprivation in animals [35] and functional MRI scan results in a patient following reversal of longstanding anterior-segment ocular disease [15]. Cortex deprived of stimulation from its primary modality could become instead responsive to alternative sensory modalities [36]. We tested this notion by determining with fMRI the extent of visual cortex responsive to visual stimulation in *RPE65-*LCA patients. Given the measurable light perception in this population, we expected to find at least some cortical response to stimulation (although it is theoretically possible that brightness detection could be mediated on a subcortical basis).

**Figure 6:**
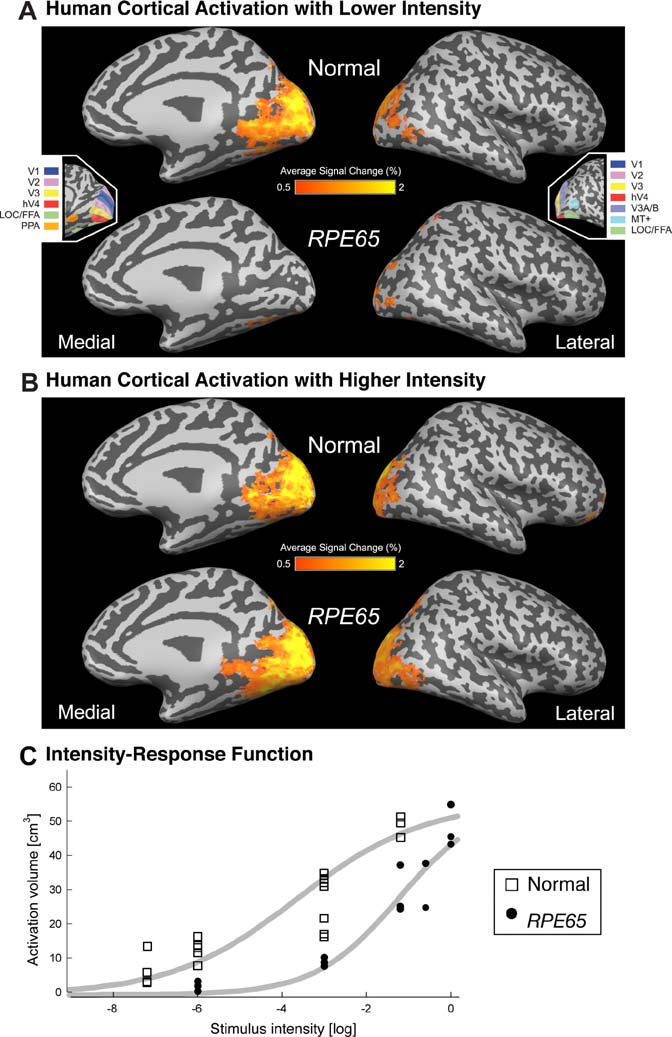
Mean Cortical Signal Change in Response to Visual Stimulation in Human RPE65-LCA (*n* = 6) and Control Populations (*n* = 8) (A and B) The BOLD fMRI response is shown for each population at two stimulus intensities: (A) −3 log and (B) at/near maximum (between −1.2 log and 0 log). The areas of response are displayed upon a digitally inflated right hemisphere. Sulci are indicated in dark gray and gyri in light gray. (Insets) The general position of several retinotopic and higher-order visual areas, derived from data from control participants, is shown. Visual area nomenclature is as published [37]. (C) Cortical activation as a function of stimulus luminance is presented. The volume of posterior cortical tissue demonstrating a substantial (>2%) response shows a sigmoidal relationship to the strength of visual stimulation in normal controls and in patients. A Hill function (gray smooth lines) is fit by eye to the data points corresponding to each participant.

First, we used a stimulus that was about 1 log unit brighter than visual threshold in *RPE65*-LCA patients and compared the cortical responses to those of visually normal individuals using the same stimulus (Figure 6A; Table 3). Data from 28 scans (56 cortical hemispheres) were combined across participants. Control individuals showed a large extent of posterior occipital and temporal cortex that had a response (>0.5% BOLD fMRI signal change) to the visual stimulus. This activation corresponds to the anatomical location of the retinotopically organized early visual areas, as well as higher-order visual association cortex with coarse retinotopic organization (e.g., form-responsive visual areas such as the lateral occipital complex [37]). In the *RPE65*-LCA patient group a greatly attenuated response, both in extent and intensity, was seen. Only small patches of responsive voxels were present within more distal (presumably peripheral) portions of early visual areas. A whole-brain random-effects analysis of these data confirmed significant differences in amplitude of response throughout the posterior visual areas (unpublished data).

**Table 3:**
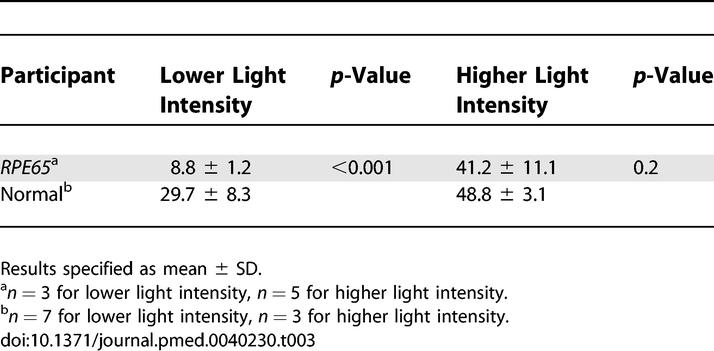
Human fMRI Results for Cortical Activation Volume (cm^3^)

The finding of reduced activation to this stimulus prompted the question of whether this was the limit of visually responsive cortex. A more suprathreshold stimulus was then used. Data from 19 scans (38 cortical hemispheres) were combined (Figure 6B; Table 3). For control participants, the extent of response was comparable to that seen for the lower level of stimulus intensity. In contrast, the *RPE65*-LCA patients demonstrated markedly increased cortical activation in response to the stronger stimulus. Notably, for the stronger stimulus, *RPE65*-LCA patients showed a cortical area responsive to visual stimulation comparable to that in controls, involving not only calcarine cortex but also dorsal and ventral areas normally devoted to extrastriate visual processing. A random-effects whole-brain statistical comparison between the controls and patients did not reveal any significant map-wise differences between the groups at this stimulation level.

To characterize more precisely the cortical responses to visual input in humans with *RPE65*-LCA, we obtained fMRI measures of neural activity in response to different light intensities following dark adaptation. Prior studies have demonstrated a correspondence between BOLD fMRI response functions and psychophysical performance (e.g., in the domain of contrast sensitivity [38]). Here, the lowest level of stimulation presented was designed to be near the perceptual threshold of normal individuals and thus several log units below threshold for people with *RPE65*-LCA (Figure 4A). Figure 6C plots the volume of cortical tissue that demonstrated a robust response to visual stimulation in control individuals and *RPE65*-LCA patients. For control participants, the lowest light stimulus presented (7.2 log units below maximum 0, Figure 6C) was associated with a small but significant neural response within visual areas. The volume of cortical tissue with a substantial response to stimulation increased monotonically with stimulus intensity. In contrast cortex of patients with, *RPE65*-LCA showed no measurable neural response to lower intensity stimuli that evoked neural responses in control participants. At 3 log units below maximum intensity, cortex of *RPE65*-LCA patients showed a measurable response, although it was on average one-third the size of that in control individuals. With increasing intensity of light stimulation, the cortical response from patients with *RPE65*-LCA grew, reaching similar volumes as in normal controls at the maximum intensities used in the study. At the maximum level of stimulation achieved in each group, there was no difference in the extent of cortical response (t[7] = 1.4; *p* = 0.2).

## Discussion

Congenitally blind *RPE65*-mutant dogs recovered responses within cortical visual areas after retinal gene therapy. Recovery was present even in a dog treated at 4 y of age. These results are concordant with demonstrations of recovery at retinal and subcortical levels [1,2,39,40] and relate well to the findings of improved visual evoked potentials and simple visual behavioral tasks in dogs and mice [1,11,39–41]. RPE65 deficiency essentially causes severe binocular light attenuation to the visual system, and a comparison to the extensive literature on cortical effects of early visual deprivation is of interest (reviewed in [42,43]). Visual deprivation in animals shortly after birth leads to a dramatic reduction of visually responsive neurons within cortical visual areas. The timing and type of deprivation affects the character and severity of alteration of cortical function [42–44]. Binocular eyelid suture, which produces modest light attenuation but severe form deprivation, produces greater abnormalities in cortical physiology than an equivalent period of dark rearing [45]. The standard model is that early visual experience during a critical period of neuronal plasticity defines the response properties of cortical visual neurons, and that after this period these properties become relatively immutable [43]. The 4-log-unit reduction in light sensitivity from retinoid cycle blockade in RPE65 deficiency likely falls between dark-rearing and lid-suture experimental paradigms. The recovery we observed after retinal gene therapy suggests that the visual cortex of the *RPE65*-mutant dog remained receptive to increased visual input for over 4 y.

While there was a dramatic recovery of cortical responsiveness following gene therapy, differences in the amplitude of neural response within the lateral gyrus remained between controls and some treated animals. These differences may be attributable to the retinal location and area treated and possibly to an effect of visual deprivation. In addition to a general decline in the responses of cortical neurons, visual deprivation disrupts the normal functional architecture of visual areas, including receptive field organization, ocular dominance, and direction selectivity [45,46]. These might differ between normal and treated animals, even if cortical extent and maximal magnitude of neural responses were comparable. Although the precise functional organization of cortical visual areas following treatment was not addressed in the present work on the *RPE65*-mutant dog, it is worth noting that some animals in our study recovered responses in cortex normally devoted to extrastriate visual areas, suggesting preserved higher-level visual function.

Human *RPE65*-LCA has captured international interest as a potential target for a retinal gene therapy approach like that used in the *RPE65*-mutant dog [12,47]. It is unknown, however, whether LCA patients with severe visual loss from birth have any receptivity of cortical substrates for restored retinal input. The literature suggests that early blind patients could show markedly abnormal anatomy in the postretinal visual pathways. A diffusion tensor imaging study of early blind patients demonstrated atrophic or absent optic nerves and geniculocortical tracts [17], while a voxel-based morphometry analysis revealed atrophy of cortical gray matter in early visual areas [16]. Optic nerve diameter was no different from normal in patients with *RPE65*-LCA, and no alteration of the LGN was found. While a small reduction in occipital white matter was found in *RPE65*-LCA patients, the subtle nature of this change suggests a difference from other early blind individuals, despite sharing a severe impairment of visual perception from infancy. Our finding of relatively preserved postretinal structure may be related to the sparing of retinal ganglion cells in humans with *RPE65*-LCA, as compared to more destructive lesions affecting the neural retina, thereby preventing the anterograde transneuronal degeneration that accompanies destruction of these neurons [48].

Cortical function was also expected to be abnormal in *RPE65*-LCA, and we found abnormal cortical activation using a light stimulus that was definitely suprathreshold. Yet, this was not the limit of visually responsive cortex. Surprisingly, an increase in stimulus intensity fully activated the cortex of humans with *RPE65*-LCA. A neural luminance-response function at the cortex of patients with *RPE65*-LCA supports the notion that given sufficient light stimulation, the cortex can be activated normally. We speculate that during early life, visual input in *RPE65*-LCA patients is sufficient for cortical development. The relatively preserved retinal structure [12], limited but detectable retinoid cycle activity [49], and a range of light level exposures during infancy and childhood may be sufficient to maintain normal postretinal anatomy and function, or possibly extend cortical plasticity into adulthood. A few case reports have examined recovery of vision in adulthood following relatively late treatment of ophthalmic disease, typically lens or corneal opacities. In one particularly well-studied case, correction of anterior segment disease in adulthood resulted in limited recovery of vision and markedly reduced cortical responses to visual stimuli, particularly in extrastriate areas [15]. Indeed, persistent deficits in integrative visual function are seen even when bilateral cataracts are treated in childhood [50], although there is recent intriguing evidence that recovery of some higher-level visual function is possible with adult treatment [51].

Our results do not necessarily predict recovery of higher-level visual function. Although a normal extent of cortex responded to visual stimulation, we are unable to state if cortical organization for vision is intact beyond elementary luminance representation. It is encouraging, however, that activity within ventral cortex normally associated with form processing is present, as this was not found in the patient with severe early anterior-segment pathology, even after it was corrected [15]. In summary, the evidence for cortical functional recovery following retinal gene therapy in the *RPE65*-mutant dog, taken together with relatively preserved cortical structure and function of humans with *RPE65* mutations, provides increased optimism regarding potential for recovery of functional vision in humans with *RPE65*-LCA, whether treatment is by gene replacement, pharmacological bypass [52], or visual prostheses [53].

## Abbreviations

BOLD: blood oxygen level–dependent
ERG: electroretinogram
fMRI: functional magnetic resonance imaging
HRF: hemodynamic response function
LCA: Leber congenital amaurosis
LGN: lateral geniculate nucleus
RPE: retinal pigment epithelium
SD: standard deviation
TPLR: transient pupillary light reflex
